# The Impact of Horizontal Marking on the VisuMax Surgical Bed Headrest on the Outcomes of Myopic Astigmatism Correction With Small Incision Lenticule Extraction

**DOI:** 10.1155/joph/8431610

**Published:** 2025-03-19

**Authors:** Yun Wang, Xiaofeng Zhang, Wenwen Pan, Li Wang, Jing Lou, Yue Xu

**Affiliations:** Department of Ophthalmology, The Fourth Affiliated Hospital of Soochow University, Suzhou 215004, China

**Keywords:** astigmatism, headrest horizontal marking, small incision lenticule extraction, vector analysis

## Abstract

**Aims:** To investigate the impact of horizontal markings on the VisuMax surgical bed headrest on the accuracy of astigmatism correction in small incision lenticule extraction (SMILE).

**Methods:** This retrospective study categorized preoperative astigmatism severity into low-astigmatism (−0.25 to −1.75 D) and moderate-to-high astigmatism (−2.00 to −4.50 D). A preoperative patient fixation training regimen coupled with applying horizontal markings on the VisuMax surgical bed headrest was introduced to improve the precision of astigmatism correction. The effectiveness of SMILE was compared to femtosecond laser-assisted in situ keratomileusis (FS-LASIK) in correcting astigmatism by using Alpins vector analysis, as well as the higher-order aberrations were measured.

**Results:** This study included 170 patients (56 eyes in the low-astigmatism group and 31 eyes in the moderate-to-high astigmatism group of SMILE; 47 eyes in the low-astigmatism group and 36 eyes in the moderate-to-high astigmatism group of FS-LASIK). At 6 months postoperatively, safety and efficacy indices between SMILE and FS-LASIK showed no significant differences for either astigmatism group (*p* > 0.05). However, significant differences were observed in surgically induced astigmatism (SIA), magnitude of error (ME), and correction index (CI). A considerable difference in equivalent spherical (SE) was found in the low-astigmatism group (*p* < 0.05). No significant differences were noted in the angle of error (AE) and its absolute value (|AE|) between the two procedures (*p* > 0.05). Both techniques increased total higher-order aberrations, spherical aberration, and vertical coma, with SMILE associated with a significantly higher increase in vertical coma than FS-LASIK (*p* < 0.05).

**Conclusions:** Augmented by precise preoperative strategies, including headrest marking and fixation training, SMILE achieves astigmatism axis correction efficacy comparable to FS-LASIK. SMILE and FS-LASIK are effective and comparable in correcting moderate-to-high astigmatism, highlighting their safety, efficacy, and predictability as corrective measures for myopic astigmatism.

## 1. Introduction

The global increase in myopia prevalence and corresponding escalation in the demand for enhanced visual outcomes have spurred advancements in corneal refractive surgery techniques. Currently, the primary surgical interventions for refractive errors include femtosecond laser-assisted in situ keratomileusis (FS-LASIK) and small incision lenticule extraction (SMILE), which realizes a “flapless” mode of surgery. The advent of femtosecond laser technology has significantly advanced traditional mechanical microkeratomes used in corneal refractive surgery offering enhanced safety and precision [1]. This innovative approach substantially mitigates complications associated with mechanical flaps, such as free flaps, incomplete flaps, and variable flap thickness [2]. SMILE leverages this technology to create a lenticule within the corneal stroma, which is subsequently extracted through a minimal, approximately 2 mm peripheral incision, eliminating the need for flap lifting. Compared to LASIK, SMILE is associated with less disruption to the subepithelial nerve plexus and provokes a milder postoperative inflammatory response than LASIK [3, 4]. Extensive research corroborates the safety, efficacy, and long-term reliability of SMILE and FS-LASIK in correcting myopic astigmatism [5, 6].

Astigmatism, a vector parameter, requires a comprehensive approach that addresses both its magnitude and axis, underscoring the need for precise surgical positioning. Vector analysis is widely employed to assess the efficacy of astigmatism correction procedures [7]. Although SMILE is established as a safe, effective, stable, and predictable option [8–11], discussions persist over its ability to correct astigmatism when compared to LASIK, mainly because of SMILE's lack of an automatic iris tracking and rotational control system found in excimer laser setups [12]. Hashemi et al. [9] advocate the superiority of FS-LASIK, which incorporates an eye-tracking system, over SMILE in correcting astigmatism. Conversely, Prickett et al. [13] suggests that many perceived rotational errors in SMILE procedures could be due to nonrotational issues, such as improper patient positioning, and that eye rotation is frequently less significant than reported. Xu et al. [14] observed that for patients with high levels of astigmatism, the rotational compensation strategies in SMILE did not provide a significant benefit, highlighting that corneal marking and using a rotational vacuum fixation ring could elevate patient anxiety and increase the chance of ring dislocation. Chan et al. [15] demonstrated that meticulous management of patient positioning to avoid head rotation could improve SMILE's performance of SMILE in astigmatism correction outcomes, making it comparable to LASIK for treating high astigmatism. Therefore, precise and simple head-position adjustments during SMILE can improve astigmatism correction outcomes. In this study, we used horizontal markings on the surgical bed headrest to maintain horizontal alignment of the patient's eye during the surgical procedure. Furthermore, preoperative fixation training was intensified to bolster patient cooperation and minimize eye movement. Under these optimized conditions, the comparative effectiveness of SMILE and FS-LASIK in correcting myopic astigmatism and their respective effects on visual quality were evaluated.

## 2. Materials and Methods

### 2.1. Patients

Data were collected from patients treated for myopic astigmatism using SMILE or FS-LASIK at the Department of Ophthalmology, Fourth Affiliated Hospital of Soochow University, between June 2021 and March 2023. The selection criteria included individuals aged ≥ 18 years; myopia within −1.00 to −10.00 diopters (D) and astigmatism between −0.25 D and −4.50 D. All participants provided informed consent for the operation. Based on their preoperative astigmatism, participants were stratified into two groups: those with astigmatism under −2.00 D were categorized into the low-astigmatism group, and those with astigmatism from −2.00 D to −4.50 D were designated as the moderate-to-high astigmatism group.

### 2.2. Ethics Approval and Consent to Participate

Ethical approval and consent to participate in the study were obtained from the Ethics Committee of the Fourth Affiliated Hospital of Soochow University (ID: 231018).

### 2.3. Surgical Procedure

Patients engaged in a structured fixation training programme for 10 min daily in the 3 days preceding the operation to improve their capacity to maintain stable eye focus during the procedure. All the surgeries were performed by the same surgeon.

### 2.4. SMILE Procedure

**Figure 1:**
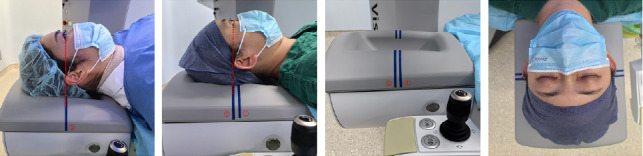
The method of headrest markers to position the head. The blue horizontal line on the headrest represents the ideal alignment of the outer canthus of the patient's eyes. The larger head is aligned with the lower horizontal Line 1, and the smaller head is aligned with the upper horizontal Line 2. As the patient lies down, the head is positioned and the horizontal lines are aligned with the outer canthus of the eyes.

The VisuMax femtosecond laser system (Carl Zeiss Meditec AG, Jena, Germany) was used for all procedures. Once the patient was positioned on the surgical bed, the surgeon meticulously adjusted the head position to ensure horizontal alignment within the headrest groove, which was facilitated by a horizontal line on the headrest intended to align with the outer canthus of the eyes. The surgical bed headrest was equipped with two horizontal lines to ensure suitability for various head sizes (Figure 1).

During the SMILE procedure, surgical parameters were adjusted based on the degree of astigmatism. In the low-astigmatism group, the following parameters were used: cap diameters, 7.58 ± 0.14 mm; cap thicknesses, 118.5 ± 6.8 μm; lenticular diameters, 6.58 ± 0.14 mm; and central lenticular thicknesses, 122.5 ± 16.8 μm. For those in the moderate-to-high astigmatism category, the following parameters were used: cap diameters, 7.61 ± 0.16 mm; cap thicknesses, 118.4 ± 6.1 μm; lenticular diameters, 6.61 ± 0.16 mm; and central lenticular thicknesses, 128.1 ± 19.1 μm. Despite these differences, both groups had a consistent side-cut angle of 90°and an incision length of 2.0 mm at 120°.

### 2.5. FS-LASIK Procedure

The FS-LASIK technique also utilizes the VisuMax femtosecond laser system to create the corneal flap, which has a standardized diameter of 8.1 mm. The flap thickness varied, averaging 104.9 μm (±5.9 μm) for the low-astigmatism group and 106.5 μm (±6.0 μm) for the moderate-to-high astigmatism group. Subsequent corneal stromal ablation was performed using an EX500 excimer laser (Alcon). The EX500 device measured the eye rotation degree with its built-in real-time iris tracking obtained during surgery and corneal topography captured by the Topolyzer before surgery. If the eye rotation is greater than 5°, reposition the head. In the low-astigmatism group, the following parameters were used: optical zone diameters, 6.56 ± 0.12 μm and ablation depths, 96.3 ± 19.2 μm. In the moderate-to-high astigmatism group, the following parameters were used: optical zone diameters, 6.56 ± 0.17 μm and ablation depths, 97.4 ± 19.2 μm.

### 2.6. Follow-Up

Follow-up assessments were comprehensive, encompassing evaluations of uncorrected distance visual acuity (UDVA), corrected distance visual acuity (CDVA), refractive changes, and corneal aberrations disclosed using Scheimpflug camera imaging (Pentacam HR; Wetzlar, Germany) to monitor recovery progress and visual improvement.

### 2.7. Statistical Analysis

Data were analyzed using SPSS statistical software (Version 25.0; Chicago, Illinois, United States of America). For normally distributed data, independent *t*-tests were used to discern significant differences between the groups. The Mann–Whitney *U* test was used for data that did not follow a normal distribution. Comparisons of preoperative and postoperative parameters within groups were performed using paired *t*-tests for normally distributed data and Wilcoxon rank-sum tests for non-normally distributed data. Statistical significance was set at a *p* value of less than 0.05.

## 3. Results

### 3.1. Preoperative Data

Fifty-six patients (56 eyes) were included in the study of the SMILE procedure for correction of low astigmatism. The mean age was 28.32 ± 6.19 years. Their preoperative metrics included spherical D, −5.43 ± 1.34 D; cylindrical D, −0.85 ± 0.38 D; and spherical equivalent (SE), −5.86 ± 1.30 D. For the moderate-to-high astigmatism group, 31 patients (31 eyes) were included, aged 25.35 ± 7.37 years, with spherical D, −4.40 ± 1.59 D; cylindrical D, −2.34 ± 0.50 D; and SE, −5.57 ± 1.54 D.

For FS-LASIK in the low-astigmatism group, 47 patients (47 eyes) were included, aged 26.77 ± 6.89 years, with spherical D, −5.85 ± 1.48 D; cylindrical D, −1.01 ± 0.50 D; and SE, −6.35 ± 1.55 D. The moderate-to-high astigmatism group included 36 patients (36 eyes), aged 23.03 ± 4.64 years, with spherical D, −4.69 ± 1.89 D; cylindrical D, −2.53 ± 0.55 D; and SE, −5.95 ± 1.84 D.

A comparison of preoperative parameters between the two groups for SMILE and FS-LASIK revealed no significant differences in age, spherical D, cylindrical D, or SE (*p* > 0.05).

### 3.2. Visual Acuity and Refraction

At 6-month follow-up, the SMILE low-astigmatism group reported a logMAR visual acuity of −0.06 ± 0.04, and the moderate-to-high astigmatism group showed −0.05 ± 0.06, which were −0.05 ± 0.06 and −0.03 ± 0.05, respectively, for the FS-LASIK groups. Statistical analysis indicated no significant differences between SMILE and FS-LASIK for the low-astigmatism and moderate-to-high astigmatism groups (*p* > 0.05).

At 6 months postoperatively, the SE for the SMILE low-astigmatism group was 0.00 ± 0.26, whereas it was 0.09 ± 0.34 for those with moderate-to-high astigmatism. For FS-LASIK, the SE was 0.16 ± 0.22 and 0.07 ± 0.27, respectively. SE was significantly lower in the SMILE group than in the FS-LASIK low-astigmatism group (*p*=0.003). However, no significant difference was observed within the moderate-to-high astigmatism group (*p* > 0.05) (Table 1).

### 3.3. Efficacy and Safety

**Figure 2:**
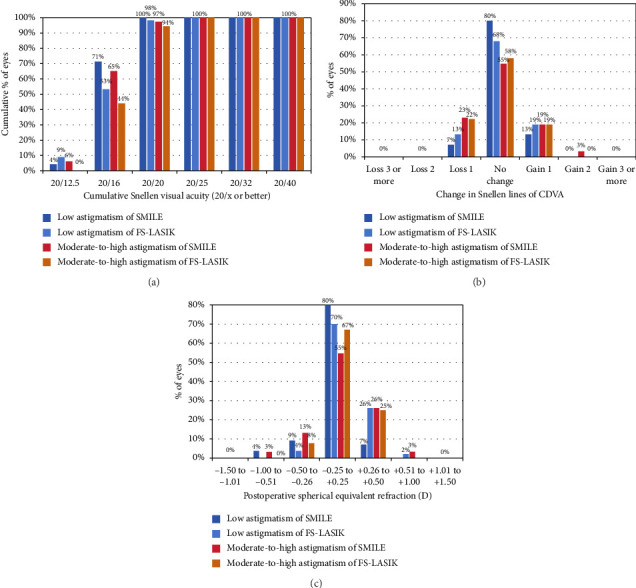
Visual outcomes for SMILE and FS-LASIK in the low-astigmatism group and the moderate-to-high astigmatism group at 6 months postoperatively. (a) Uncorrected distance visual acuity. (b) Change in corrected distance visual acuity. (c) Spherical equivalent refraction accuracy.

The effectiveness index, defined as the ratio of postoperative UDVA to preoperative BCVA, showed that in the SMILE low-astigmatism group, 100% (56 eyes) achieved a UDVA of 20/20 compared to 97% (30 eyes) in the moderate-to-high astigmatism group. In the FS-LASIK group, 98% (46 eyes) of the low-astigmatism group and 94% (34 eyes) of the moderate-to-high astigmatism group achieved UDVA of 20/20 (Figure 2(a)). The effectiveness index was 1.00 ± 0.11 for the SMILE low-astigmatism group and 0.99 ± 0.17 for the moderate-to-high astigmatism group, with FS-LASIK groups demonstrating similar indices (1.00 ± 0.13 and 0.98 ± 0.14, respectively). There were no significant differences in effectiveness between SMILE and FS-LASIK across the astigmatism groups (*p* > 0.05).

The safety index, or the ratio of postoperative CDVA to preoperative CDVA, revealed that in the SMILE group, 7% (4 eyes) of the low-astigmatism group and 23% (7 eyes) of the moderate-to-high astigmatism group experienced a loss of ≥ 1 line of CDVA. FS-LASIK exhibited a comparable trend, with 13% (6 eyes) in the low-astigmatism group and 22% (8 eyes) in the moderate-to-high astigmatism group losing ≥ 1 line of CDVA. None of the participants lost more than two lines of CDVA at 6 months postoperatively (Figure 2(b)). At this 6 month mark, the safety index was 1.02 ± 0.09 for the SMILE low-astigmatism group and 1.02 ± 0.16 for the moderate-to-high astigmatism counterparts, which is slightly higher than FS-LASIK (1.01 ± 0.11 and 1.00 ± 0.12, respectively). No significant difference in safety outcomes was observed between the SMILE and FS-LASIK procedures in both the low- and moderate-to-high astigmatism groups (*p* > 0.05) (Table 1).

### 3.4. Stability

Among the SMILE low-astigmatism group, 96% (54 eyes) maintained a postoperative SE within ±0.50 D, and 94% (29 eyes) achieved the same in the moderate-to-high astigmatism group. A more stringent criterion of ±0.25 D showed that 80% (45 eyes) in the low-astigmatism group and 55% (17 eyes) in the moderate-to-high group met this standard. Statistical analysis indicated comparable refractive stability between FS-LASIK and SMILE, with FS-LASIK showing 98% (46 eyes) in the low-astigmatism group and 100% (36 eyes) of those in the moderate-to-high maintaining a postoperative SE within ±0.50 D. Within the ±0.25 D criterion, 70% (33 eyes) of the low-astigmatism group and 67% (24 eyes) of the moderate-to-high group achieved this level of refractive precision.  Figure 2(c) illustrates the refractive outcomes at 6 months postoperatively for each group.

### 3.5. Vector Analysis

**Figure 3:**
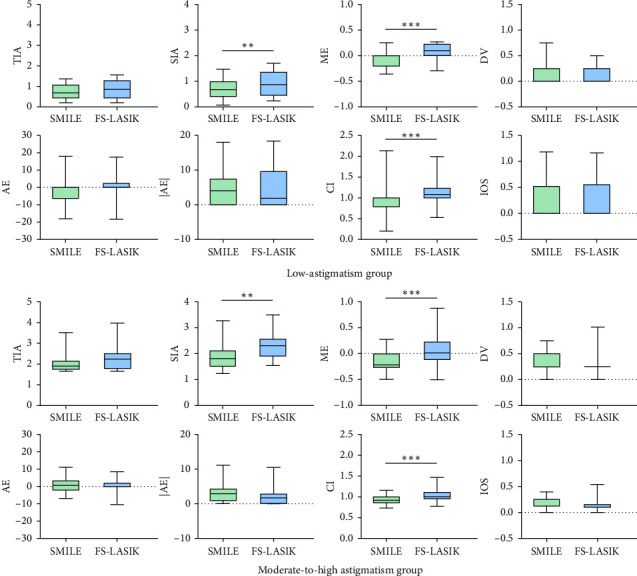
Astigmatism vector of SMILE and FS-LASIK in the low-astigmatism group and the moderate-to-high astigmatism group at 6 months postoperatively. Data are expressed as mean ± standard deviation (⁣^∗∗^*p* < 0.01 and ⁣^∗∗∗^*p* < 0.001).

The vector analysis comparing SMILE and FS-LASIK outcomes at 6 months postoperatively is summarized in Figure 3. The analysis highlighted no significant differences in target-induced astigmatism (TIA), difference vector (DV), angle of error (AE), absolute value of AE (|AE|), or index of success (IOS). However, the SMILE group demonstrated a significantly lower surgically induced astigmatism (SIA) than the FS-LASIK group (*p* < 0.01). The magnitude of error (ME), calculating the difference between SIA and TIA, indicated a tendency toward undercorrection in SMILE (−0.05 ± 0.16 and −0.16 ± 0.19 for low- and moderate-to-high astigmatism groups, respectively) and a slight overcorrection in FS-LASIK (0.09 ± 0.02 and 0.07 ± 0.26, respectively), with these differences being significant (*p* < 0.001). The correction index (CI), representing the ratio of SIA to TIA, underscored this trend with SMILE showing undercorrection (CI of 0.94 ± 0.29 and 0.92 ± 0.10, respectively) and FS-LASIK indicating overcorrection (CI of 1.13 ± 0.04 and 1.04 ± 0.12, respectively), both differences being significant (*p* < 0.001).

**Figure 4:**
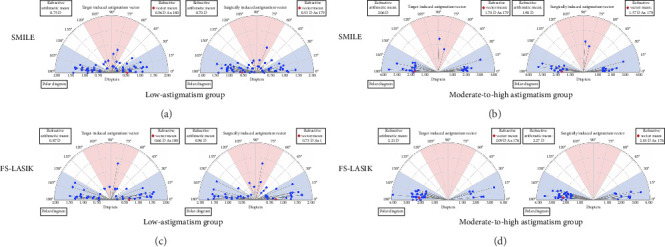
Single-angle polar plots for the target-induced astigmatism (TIA) vector and surgically induced astigmatism (SIA) vector at 6 months postoperatively after SMILE in the low-astigmatism group (a) and the moderate-to-high astigmatism group (b) and FS-LASIK in the low-astigmatism group (c) and the moderate-to-high astigmatism group (d).

**Figure 5:**
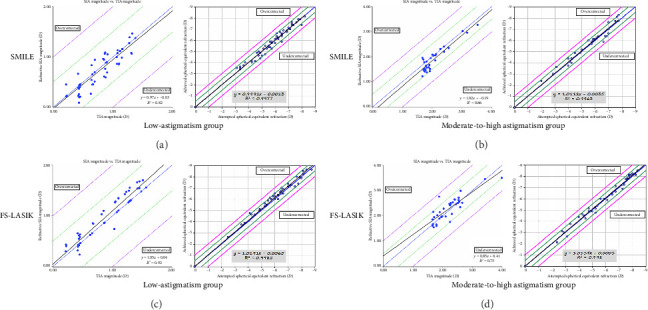
Scatter plot of the target-induced astigmatism vector versus surgically induced astigmatism vector and spherical equivalent refraction attempted versus achieved at 6 months postoperatively after SMILE in the low-astigmatism group (a) and the moderate-to-high astigmatism group (b) and FS-LASIK in the low-astigmatism group (c) and the moderate-to-high astigmatism group (d).

Figures 4 and 5 depict the relationship between the TIA and SIA across the groups examined.  Figure 4 shows coordinate plots of the postoperative TIA versus SIA for each group, while Figure 5 presents scatter plots comparing TIA to SIA at the 6-month postoperative mark. For the SMILE procedure, trend lines for the low- and moderate-to-high astigmatism groups were plotted below the diagonal, signifying a tendency toward undercorrection of astigmatism. Conversely, the trend line was above the diagonal for the FS-LASIK low-astigmatism group, suggesting an overcorrection of astigmatism, which shifted below the diagonal for the moderate-to-high astigmatism group.

### 3.6. Corneal Aberration

**Figure 6:**
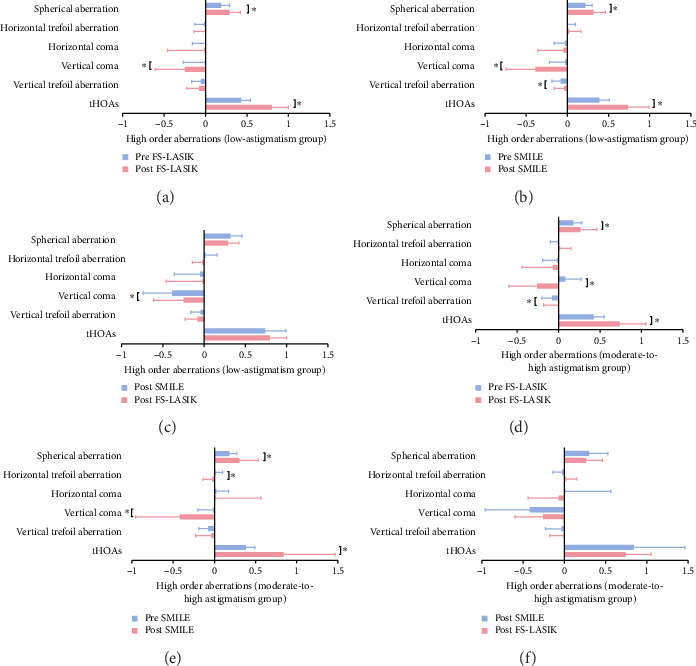
Comparison of high order aberrations before and after FS-LASIK (a), (d) and SMILE (b), (e) and between SMILE and FS-LASIK (c), (f) in the low- and moderate-to-high astigmatism group. Data are expressed as mean ± standard deviation (⁣^∗^*p* < 0.05).

A comparative analysis of corneal aberrations revealed no significant differences between the SMILE and FS-LASIK groups preoperatively, encompassing both low- and moderate-to-high astigmatism groups, in terms of preoperative total higher-order aberrations, vertical coma, vertical trefoil, horizontal coma, horizontal trefoil, and spherical aberration (*p* > 0.05). However, 6 months postoperatively, there was an observed increase in total higher-order aberrations, spherical aberration, and vertical coma across all groups (*p* < 0.05). The low-astigmatism group exhibited a significant difference in vertical coma between SMILE (−0.39 ± 0.35) and FS-LASIK (−0.25 ± 0.36) groups (*p* < 0.05). Other corneal aberrations within the low-astigmatism group and all corneal aberrations within the moderate-to-high astigmatism group did not show significant postoperative differences between the SMILE and FS-LASIK techniques (*p* > 0.05), as depicted in Figure 6.

## 4. Discussion

Extensive research has validated the high efficacy, safety, and predictability of SMILE and FS-LASIK in correcting myopic astigmatism [9, 16, 17]. This study reinforces these findings, demonstrating that incorporating horizontal markings on the surgical bed headrest and advanced preoperative fixation training significantly contribute to optimal surgical outcomes. Remarkably, 100% of the patients in the SMILE low-astigmatism group and 97% in the moderate-to-high astigmatism group achieved a UDVA of 20/20 or better at 6 months postoperatively. Patients with FS-LASIK showed comparable success rates, with 98% in the low-astigmatism group and 94% in the moderate-to-high astigmatism group, reaching similar UDVA milestones. Consistent with Zhou et al. [18], no participants experienced a loss of more than one line of CDVA. The safety indices for all groups exceeded 1, and the efficacy indices were above one in the low-astigmatism group and close to one in the high-astigmatism group, signifying no substantial difference in the effectiveness and safety between SMILE and FS-LASIK when employing a combination of horizontal headrest marking and preoperative fixation training. Predictability was commendable, with 96% of SMILE and 98% of FS-LASIK participants in the low-astigmatism group achieving a postoperative SE within ±0.50 D, extending to 94% for SMILE and 100% for FS-LASIK in the moderate-to-high astigmatism groups. The observed variance in SE between SMILE and FS-LASIK in the low-astigmatism cohort suggests the potential for undercorrection in SMILE and overcorrection in FS-LASIK for low levels of astigmatism. However, both methods exhibited undercorrection in the moderate-to-high astigmatism group, with no significant difference in SE outcomes.

Concerns regarding SMILE primarily focus on its propensity for undercorrecting astigmatism, which is attributed to intraoperative eye rotation and the absence of eye-tracking capabilities [16, 19]. The challenge of compensating for eye movements during surgery has prompted researchers to advocate manual marking techniques to diminish astigmatism errors [20–22]. However, some scholars caution against the potential drawbacks of corneal marking, arguing that it may exacerbate patient anxiety and increase the risk of suction loss during the procedure [23, 24]. Despite the minor static cyclotorsion induced by moving from an upright to a supine position, research indicates that such rotation remains minimal. Emphasizing rigorous control over the patient's head and body position during surgery is essential to minimize the impact of eye rotation on surgical outcomes [15]. Previous research highlighted that 82% of patients experience cyclotorsion when transitioning from sitting to lying positions, with 86% of eyes with high astigmatism rotating up to 5° and none exceeding 10° [23]. Moreover, studies have identified several drawbacks associated with manual corneal marking. Primary concerns include the inherent accuracy of the corneal marking process and the increased risk of ring dislocation when the contact ring is rotated after suction. Andreas et al. [25] observed a marking error of 2.80 ± 1.88° using slit-lamp horizontal corneal markings. Woo et al. [26] reported marking errors of 6.01 ± 2.62° under the slit lamp, 5.79 ± 2.70° through the surgeon's unaided observation, and 3.77 ± 1.99° using a horizontal pendulum. These findings suggest that manual marking introduces an error margin of 3.8°–6.0°, which is approximately equivalent to the natural rotation of the eye, casting doubt on the efficacy of corneal marking as a corrective strategy. Prickett et al. [13] suggested that some reported instances of eye rotation might include nonrotational factors such as the patient's supine posture during the procedure. Li et al. [27] confirmed that using laser cross-hair for patient positioning could prevent head rotation and improve astigmatism correction using the SMILE technique. However, the laser cross-hair system setup presents challenges and complexities in the clinical practice. This study did not use corneal marking; it focussed on minimizing correction errors due to eye rotation and tilt by enhancing preoperative fixation training and applying horizontal markers to the surgical bed headrest for patient alignment.

Astigmatism is analyzed as a vector parameter encompassing both magnitude and axis. Using the Alpins method for postoperative assessment, significant differences in SIA, ME, and CI were observed between SMILE and FS-LASIK in the low-astigmatism cohort. SMILE exhibited a CI < 1, indicating an undercorrection trend for low astigmatism, whereas FS-LASIK's CI > 1 suggested a tendency toward overcorrection. Furthermore, a substantial difference in SE was observed at 6 months postoperatively. Given the surgical goal of slight postoperative overcorrection, FS-LASIK was deemed more efficacious in correcting low myopic astigmatism, consistent with previous research findings [15]. In our study, we classified astigmatism ranging from −0.25 D to −1.75 D as the low-astigmatism group. Although the low levels of astigmatism may have limited the results, we included these cases to provide a more comprehensive understanding of the range of astigmatism treated. In the moderate-to-high astigmatism group, FS-LASIK CI of 1.04 indicated a 4% overcorrection.  Figure 5(d) depicts the pattern of transitioning from overcorrection to undercorrection, with the turning point around −2.50 D, mirroring findings from earlier FS-LASIK studies, which noted overcorrection in low astigmatism and undercorrection in higher degrees of astigmatism [28]. Contrary to several studies [9, 19, 24] that found postoperative CI for LASIK in high-astigmatism groups to be < 1, this study included astigmatism of ≥ −2.00 D as moderate-to-high, differing from others such as Zhang [19], who considered ≥ −2.50 D. In the moderate-to-high astigmatism group, SMILE still showed a tendency toward undercorrection of astigmatism. Similarly, a study by Zhang [19] also demonstrated that both SMILE and FS-LASIK exhibited insufficient correction when addressing high astigmatism. However, no significant difference in SE was detected in the moderate-to-high astigmatism group postoperatively between the two methods, with both showing slight overcorrection of refractive errors. In summary, by integrating fixation training with horizontal marking on surgical bed rest, this study concluded that SMILE and FS-LASIK yield comparable results in treating moderate-to-high myopic astigmatism, offering a nuanced approach to correction.

The axis is pivotal in astigmatism correction [20, 23, 24], as illustrated by Alpin's vector analysis, demonstrating that a mere 4° deviation from the intended axis could theoretically lead to 14% undercorrection of astigmatism. In contrast, a deviation of 15° could result in an undercorrection exceeding 50%. This study recorded the AE for all groups within a 3° margin, showing no significant difference in terms of AE and |AE| between SMILE and FS-LASIK. These findings indicate minimal postoperative astigmatic axis deviation. Despite SMILE's lack of an eye-tracking feature, the study effectively minimized eye rotation through comprehensive preoperative fixation training and precise head positioning, facilitated by marking with headrest, revealing no difference in astigmatic axis changes between SMILE and FS-LASIK. Furthermore, the study observed that |AE| was higher in the low-astigmatism group than in the moderate-to-high astigmatism group, consistent with the findings from previous studies [29, 30]. This observation could be attributed to decreased sensitivity to low levels of astigmatism among patients, complicating the precise determination of the axis and leading to a higher propensity for errors in astigmatic axis determination, an issue that diminishes with increasing astigmatism severity.

Corneal refractive surgery corrects refractive errors by reshaping the corneal stroma. However, this modification alters the innate aspheric shape, reducing its natural aberration-mitigation capabilities and increasing postoperative corneal aberrations. This study corroborates previous findings by Zhao et al. [21] indicating a postoperative increase in total higher-order aberrations, spherical aberration, and vertical coma in both SMILE and FS-LASIK procedures. The surge in postoperative aberrations is attributed to corneal shape modifications, variability in laser energy deployment, tear film instability, and corneal healing responses [31]. The increase in postoperative vertical coma was significantly higher in the SMILE group than in the FS-LASIK in the low-astigmatism group, potentially because of the superior incision characteristic of the SMILE procedure [21], indicating FS-LASIK better than SMILE at correcting low astigmatism. No significant difference was observed within the moderate-to-high astigmatism group between FS-LASIK and SMILE. Payne et al. [32] have noted an association between the escalation of vertical coma and decentration, particularly in the vertical direction. However, the association between the direction of decentration and coma type remains controversial and warrants further investigation.

The present study has a few limitations. One limitation of this study is its retrospective design, which may introduce selection bias. Nevertheless, our results align with previous prospective studies demonstrating comparable efficacy between SMILE and FS-LASIK in astigmatism correction. For instance, Hashemi et al. [9] reported no significant difference in the CI between SMILE and FS-LASIK for myopic astigmatism. In addition, Yang et al. [20] showed that with appropriate preoperative alignment strategies, SMILE can achieve equivalent astigmatic correction even in cases of high astigmatism. These findings suggest that even in a prospective study, SMILE would not be inferior to FS-LASIK in astigmatism correction. Nonetheless, future randomized controlled trials are necessary to further validate these observations. Another point is that the follow-up time of this study was 6M after surgery, and the comparison of the postoperative astigmatism correction between SMILE and FS-LASIK needs further long-time study. Also, we did not measure the eye rotation angle in SMILE. In consequence, for individual patients with significant static cyclotorsion angles in SMILE, it may significantly impact the effectiveness of astigmatism correction. Finally, there is anticipation for the future development of SMILE technology, particularly the incorporation of eye-tracking, which promises to enhance precision in astigmatism correction and offer improved patient visual outcomes.

In summary, integrating fixation training and horizontal markings on the surgical bed headrest resulted in comparable astigmatism axis correction accuracy between SMILE and FS-LASIK. When addressing astigmatic refractive correction, SMILE tends to result in an overall slight undercorrection. In contrast, FS-LASIK performed better at correcting low astigmatism, although both methods yielded similar outcomes in managing high astigmatism. SMILE and FS-LASIK demonstrated good safety, efficacy, and predictability for correcting myopic astigmatism.

## Figures and Tables

**Table 1:** Comparative postoperative visual outcomes of SMILE and FS-LASIK^a^.

Parameters	Low-astigmatism groups			Moderate-to-high astigmatism groups		
	FS-LASIK	SMILE	*p* value	FS-LASIK	SMILE	*p* value
SE (D)	0.16 ± 0.22	0.00 ± 0.26	0.003⁣^∗^	0.07 ± 0.27	0.09 ± 0.34	0.607
UDVA (logMAR)	−0.05 ± 0.06	−0.06 ± 0.04	0.144	−0.03 ± 0.05	−0.05 ± 0.06	0.069
Effectiveness index	1.00 ± 0.13	1.00 ± 0.11	0.975	0.98 ± 0.14	0.99 ± 0.17	0.957
Safety index	1.01 ± 0.11	1.02 ± 0.09	0.872	1.00 ± 0.12	1.02 ± 0.16	0.922

^a^Values are expressed as mean ± standard deviation.

⁣^∗^*p* < 0.05.

## Data Availability

The data that support the findings of this study are available from the corresponding author upon reasonable request.
